# Health-related quality of life with KDQOL-36 and its association with self-efficacy and treatment satisfaction in Korean dialysis patients

**DOI:** 10.1007/s11136-012-0203-x

**Published:** 2012-05-26

**Authors:** Jong-Yeon Kim, Bokyoung Kim, Ki-Soo Park, Ji-Young Choi, Jung-Ju Seo, Sun-Hee Park, Chan-Duck Kim, Yong-Lim Kim

**Affiliations:** 1 Department of Preventive Medicine, Daegu Catholic University School of Medicine, Daegu, Korea; 2 Department of Preventive Medicine, Institute of Health Sciences, Gyeongsang National University School of Medicine, 816-15 Jinjudaero, Jinju city, Gyeongnam Korea; 3 Department of Internal Medicine, Kyungpook National University School of Medicine, Daegu, Korea; 4 Clinical Research Center for End Stage Renal Disease, Daegu, Korea

**Keywords:** ESRD, Quality of life, Self-efficacy, Treatment satisfaction

## Abstract

**Background and objectives:**

This study was conducted to measure the level of health-related quality of life (HRQOL) and to reveal the association of self-efficacy and treatment satisfaction with it in Korean dialysis patients.

**Design, setting, participants, and measurements:**

The study subjects were 237 patients receiving either hemodialysis (HD) or peritoneal dialysis (PD) from two university hospitals, from February to June in 2010. We investigated HRQOL using the Korean version of Kidney Disease Quality of Life Short Form 36 (KDQOL-36), and self-efficacy and treatment satisfaction by self-administrative questionnaire and their dialysis-related variables by reviewing clinical records. The associations of self-efficacy and treatment satisfaction with HRQOL were assessed using multiple linear regression analysis.

**Results:**

The mean HRQOL results were as follows: Physical component score (PCS) was 39.1 ± 8.5, Mental component score (MCS) 44.6 ± 6.8, symptom/problem list was 67.6 ± 17.1, effects of disease score was 58.5 ± 19.6, and burden of disease score was 41.1 ± 28.4. Between PD and HD patients, we could find significant difference only in the symptom/problem list. After removing confounder’s effects by multivariate analysis, respectively, treatment goal self-efficacy and treatment management self-efficacy were significantly related with all 5 domains, except PCS. Treatment satisfaction was significantly related with PCS, MCS, and effects of kidney disease.

**Conclusions:**

Patients’ self-efficacy and treatment satisfaction could influence their HRQOL. Regular and systematic monitoring using KDQOL-36 and interventions to increase self-efficacy and treatment satisfaction should be considered in dialysis care in Korea.

## Introduction

In the recent decades, health-related quality of life (HRQOL) has been widely accepted as a valid marker of both treatment outcome and mortality for patients with chronic diseases [1, 2]. It is also considered as a consistent and powerful predictor of health outcomes in end stage renal disease (ESRD) [3–9]. It is generally agreed that HRQOL of dialysis patients is usually poorer than that of the age-matched subjects from the general population, because of the typically high burden of comorbidity and complications of ESRD [3].

Recently, there has been increasing interest in factors impacting on HRQOL of ESRD patients, but it has not been clearly confirmed yet which of them showed the strongest influence on the patients’ HRQOL [9–13]. Generally, ESRD patients are in great need for self-management of long-term illnesses and must frequently make daily decisions involving fluid intake, nutrition, physical activity, and symptom management [13–15]. Additionally, self-efficacy and treatment satisfaction are known to influence these self-management and decision-making processes [16–18]. Until now, several previous studies have dealt with self-efficacy [19–22] and treatment satisfaction [23, 24], but most of them used SF-36 and showed limitation in considering disease-specific HRQOL.

Therefore, the purpose of this study was to measure the level of HRQOL using KDQOL-36 and to reveal the association of self-efficacy and treatment satisfaction with HRQOL in Korean patients with ESRD.

## Subjects and methods

### Subjects

The prevalent dialysis patients, including both hemodialysis (HD) and peritoneal dialysis (PD) patients from two university hospitals, had been included from February to June in 2010. Patients who were on the dialysis for acute kidney injury were excluded from the study. The Institutional Review Board of Kyungpook National University Hospital approved the research protocol [IRB Number: KNUH_09_1045]. All subjects gave written informed consent before study enrollment.

### Instruments

We investigated the socio-demographic factors, treatment satisfaction, self-efficacy and HRQOL by self-administrative questionnaire and dialysis-related factors by reviewing the clinical records.

Self-efficacy was composed of 11-items with 2 subscales (i.e., 7 items for treatment goal self-efficacy and 4 items for treatment management self-efficacy). The items were modified from those referring to empowerment of diabetes patients [18]. Responses were on 5-point Likert scales; we used mean scores (range: 1–5). The modified scale used in this study demonstrated adequate content validity and internal consistency reliability (Cronbach’s alpha = 0.90 and 0.86, respectively). Construct validity was supported by factor analysis, and the result was that self-efficacy was composed of two subscales.

The 10-item questionnaire asked about treatment satisfaction during dialysis. Responses were on a 7-point Likert scale where 0—strongly disagree and 6—strongly agree. Scores were summed and ranged from 0 to 60. In this study, the internal consistency coefficient (Cronbach’s alpha) was 0.88 and factor analysis resulted in all items loading greater than 0.42 on a single factor.

The Korean version of the KDQOL-36 includes 12 items that provide a generic chronic disease core (i.e., the SF-12, a shorter version of the SF-36), as well as 24 additional items (i.e., kidney-disease-targeted items). The 24 additional items focus on particular health-related concerns of individuals with kidney disease (i.e., symptom/problem list, 12 items; effects of kidney disease, 8 items; and burden of disease, 4 items). The item scores were aggregated without weighting and transformed linearly to a 0–100 possible range, with higher scores indicating better states, which resulted in a total of dimensions.

### Statistical analysis

To compare scores of HRQOL subscales by socio-demographic and disease-related factors, the Student’s t test and analysis of variance were used. The associations of self-efficacy and treatment satisfaction with HRQOL subscales were assessed using Pearson correlation coefficients. To assess these associations after adjusting for possible confounders, multiple linear regression analyses were performed. Selected confounders were age, sex, educational level, job, dialysis method, dialysis duration, causes of ESRD, dialysis adequacy, comorbidity, and serum albumin level. The SPSS 15.0 statistical package for Windows (SPSS, Chicago, IL) was used for all statistical analyses.

## Results

A total of 237 patients were recruited in this study, including 172 (72.6 %) HD patients and 65 (27.4 %) PD patients.

Their socio-demographic and disease-specific characteristics are shown in Tables 1 and 2, respectively. Overall, 54.0 % were male, 27.8 % younger than 49 years, 24.9 % 50–59 years, 26.2 % 60–69 years, and 21.1 % older than 70 years. In the disease-specific characteristics, 61.0 % received dialysis treatment for more than 2 years and diabetes mellitus was the most common (39.7 %) cause of ESRD, followed by hypertension (29.1 %) and glomerulonephritis (10.5 %).

**Table 1:** Health-related quality of life (HRQOL) scores by general characteristics (unit: mean ± SD)

	*N* ( %)	Physical component summary	Mental component summary	Symptom/problem list	Effect of disease	Burden of disease
*Sex*						
Male	128 (54.0)	40.3 ± 9.4^*^	45.3 ± 6.2	68.8 ± 17.2	58.7 ± 19.6	40.3 ± 28.0
Female	109 (46.0)	37.8 ± 9.6	43.9 ± 7.4	66.1 ± 17.0	58.3 ± 19.6	42.1 ± 29.0
*Age*						
<49 years	66 (27.8)	42.3 ± 8.9^**^	44.1 ± 6.6	67.4 ± 19.5	58.3 ± 19.1	40.7 ± 19.1
50–59 years	59 (24.9)	40.1 ± 10.1	43.9 ± 7.7	69.4 ± 16.3	59.9 ± 20.8	43.6 ± 29.7
60–69 years	62 (26.2)	38.8 ± 9.1	45.4 ± 6.4	68.0 ± 15.2	57.6 ± 20.5	43.3 ± 28.8
+70 years	50 (21.1)	34.1 ± 9.5	45.2 ± 6.6	64.9 ± 17.2	58.2 ± 18.0	36.5 ± 28.4
*Educational level*						
Elementary or under	52 (21.9)	36.6 ± 9.7^**^	43.6 ± 7.4	66.0 ± 14.7	60.0 ± 19.3	45.9 ± 31.3
Middle school	50 (21.1)	37.0 ± 10.4	45.7 ± 6.1	63.8 ± 18.2	56.1 ± 22.8	45.0 ± 30.8
High school	80 (33.8)	40.5 ± 8.9	43.9 ± 7.3	68.2 ± 17.8	57.8 ± 18.5	42.0 ± 28.0
College or over	55 (23.2)	41.5 ± 9.5	45.7 ± 6.0	71.6 ± 16.9	60.2 ± 18.5	31.8 ± 21.7
*Job*						
Yes	68 (28.7)	41.9 ± 8.7^**^	44.3 ± 6.5	70.4 ± 17.6	60.6 ± 18.7	37.6 ± 24.9
No	169 (71.3)	38.0 ± 9.6	44.7 ± 7.0	66.4 ± 16.9	57.6 ± 19.9	42.5 ± 29.7
Total	237 (100.0)	39.1 ± 9.5	44.6 ± 6.8	67.6 ± 17.1	58.5 ± 19.6	41.1 ± 28.4

* *p* < 0.05, ** *p* < 0.01

**Table 2:** HRQOL scores by disease-specific characteristics (unit: mean ± SD or Pearson’s correlation coefficient)

	*N* ( %)	Physical component summary	Mental component summary	Symptom/problem list	Effect of disease	Burden of disease
*Dialysis type*						
HD	172 (72.6)	39.3 ± 9.7	44.6 ± 7.0	69.6 ± 16.6^**^	59.5 ± 19.4	39.9 ± 27.7
PD	65 (27.4)	38.7 ± 9.0	44.8 ± 6.4	62.1 ± 17.5	55.9 ± 20.0	44.3 ± 30.1
*Dialysis duration*						
<2 years	93 (39.4)	40.0 ± 9.7	44.0 ± 6.7	69.5 ± 16.7	58.6 ± 19.6	40.9 ± 28.3
2–5 years	82 (34.7)	39.0 ± 9.6	45.0 ± 7.1	68.0 ± 17.5	59.3 ± 19.9	43.0 ± 29.6
>5 years	62 (26.3)	38.0 ± 9.1	45.1 ± 6.7	64.0 ± 16.6	57.3 ± 19.5	39.1 ± 27.3
*Cause of ESRD*						
Hypertension	69 (29.1)	38.2 ± 9.8	45.4 ± 6.0	70.6 ± 14.9	61.5 ± 18.9	52.5 ± 32.9^**^
Diabetes	94 (39.7)	39.0 ± 9.1	45.5 ± 7.5	65.2 ± 17.7	57.7 ± 20.3	34.7 ± 24.1
Glomerulonephritis	25 (10.5)	42.6 ± 11.3	42.4 ± 7.7	73.2 ± 18.1	59.5 ± 18.8	48.2 ± 29.9
Others	49 (20.7)	38.9 ± 8.8	43.0 ± 5.6	65.1 ± 17.6	55.2 ± 19.6	43.7 ± 22.7
*Dialysis adequacy*						
Adequate	171 (72.2)	38.6 ± 9.6	44.4 ± 7.0	68.0 ± 17.4	57.4 ± 20.0	41.1 ± 29.5
Inadequate	39 (27.8)	40.4 ± 9.3	45.3 ± 6.5	66.4 ± 16.5	61.4 ± 18.4	41.1 ± 25.5
*Co*-*existence of AMI*						
Yes	5 (2.1)	37.6 ± 5.4	50.0 ± 3.4	63.3 ± 18.2	45.6 ± 18.0	41.2 ± 29.2
No	232 (97.9)	39.2 ± 9.6	44.5 ± 6.8	67.3 ± 17.1	58.8 ± 19.6	41.1 ± 28.5
*Co*-*existence of Stroke*						
Yes	22 (9.3)	33.1 ± 9.5^**^	46.7 ± 5.8	62.9 ± 16.2	57.2 ± 18.5	52.6 ± 32.4
No	215 (90.7)	39.7 ± 9.4	44.4 ± 6.9	68.0 ± 17.2	58.6 ± 19.7	39.9 ± 27.8
*Co*-*existence of HTN*						
Yes	199 (84.0)	38.8 ± 9.3	44.6 ± 6.8	67.4 ± 16.6	57.1 ± 18.7	42.2 ± 28.3
No	38 (16.0)	40.7 ± 10.7	44.5 ± 6.6	68.4 ± 19.8	65.9 ± 22.7	35.2 ± 28.5
*Co*-*existence of IHD*						
Yes	16 (6.8)	37.2 ± 9.4	47.7 ± 5.7	71.5 ± 10.8	65.4 ± 17.9	57.4 ± 28.3
No	221 (93.2)	39.3 ± 9.4	44.4 ± 6.9	67.3 ± 17.5	58.0 ± 19.6	39.9 ± 28.1
*Co*-*existence of DM*						
Yes	119 (50.2)	36.8 ± 8.8^**^	45.3 ± 6.1	67.1 ± 15.6	56.9 ± 19.4	43.6 ± 30.7
No	118 (49.8)	41.5 ± 9.7	44.0 ± 7.4	68.0 ± 18.7	60.1 ± 19.7	38.6 ± 25.8
*Number of co*-*existing disease*						
None	25 (10.5)	41.9 ± 10.9^**^	45.5 ± 6.7	70.8 ± 18.9	61.1 ± 22.0	37.7 ± 26.2^**^
1 Disease	92 (38.8)	41.7 ± 9.3	43.0 ± 7.6	68.1 ± 18.9	60.1 ± 20.0	39.1 ± 27.3
2 Diseases	96 (40.5)	37.2 ± 8.5	45.3 ± 6.1	66.0 ± 15.9	55.1 ± 18.4	38.0 ± 27.9
+3 Diseases	24 (10.1)	34.0 ± 9.3	47.4 ± 5.6	68.2 ± 12.4	59.0 ± 18.6	64.6 ± 27.9
Serum albumin level^a^	3.76 ± 0.46^a^	0.221^**^	-0.097	0.220^**^	0.052	-0.123
Total	237 (100.0)	39.1 ± 9.5	44.6 ± 6.8	67.6 ± 17.1	58.5 ± 19.6	41.1 ± 28.4

* *p* < 0.05, ** *p* < 0.01

^a^Serum albumin level was expressed as “mean ± SD” and its association with HRQOL was analyzed by Pearson’s correlation coefficient

The mean HRQOL results in 5 domains were as follows: PCS score was 39.1 ± 9.5; MCS, 44.6 ± 6.8; symptom/problem list, 67.6 ± 17.1; effect of disease, 58.5 ± 19.6; and burden of disease, 41.1 ± 28.4 (Table 1). In PCS, female, older, less-educated, and jobless patients, with stroke or diabetes mellitus, reported significantly lower QOL than the others (*p* < 0.05). In symptom/problem list, patients in PD reported significantly lower QOL than the others (*p* < 0.01), and factors associated with burden of disease were the cause of ESRD and the number of co-existing diseases (*p* < 0.05). Serum albumin showed positive correlation with both PCS and effects of disease (*p* < 0.01). Among all patients, the mean treatment goal self-efficacy was 3.1 ± 0.6; treatment management self-efficacy, 3.2 ± 0.6; and treatment satisfaction, 32.7 ± 11.4, respectively (Table 3). Treatment goal self-efficacy showed positive correlation with PCS, symptom/problem list, effects of kidney disease, and burden of kidney disease, whereas treatment management self-efficacy correlated with symptom/problem list and effects of disease (both *p* < 0.05). Treatment satisfaction showed positive correlation with PCS and effects of disease (both *p* < 0.05).

**Table 3:** Correlation between self-efficacy, treatment satisfaction, and HRQOL (unit: Pearson’s correlation coefficient)

	Mean ± SD	Health-related quality of life				
		Physical component summary	Mental component summary	Symptom/problem list	Effect of disease	Burden of disease
Treatment goal self-efficacy (range: 1–5)	3.1 ± 0.6	0.19^**^	0.16^**^	0.23^**^	0.19^**^	0.16^*^
Treatment management self-efficacy(range: 1–5)	3.2 ± 0.6	0.12	0.22^**^	0.21^**^	0.20^**^	0.12
Treatment satisfaction (range: 0–60)	32.7 ± 11.4	0.27^**^	0.18^**^	0.12	0.28^**^	−0.13

* *p* < 0.05, ** *p* < 0.01

After adjusting for confounders, both treatment goal self-efficacy and treatment management self-efficacy significantly correlated with all 5 domains, except PCS. Treatment satisfaction was significantly related with PCS, MCS, and effects of kidney disease (Table 4).

**Table 4:** Association of self-efficacy, treatment satisfaction, and HRQOL by multiple linear regression analysis

	Physical component summary			Mental component summary			Symptom/problem list			Effect of disease			Burden of disease		
	b	SE	*p* Value	b	SE	*p* Value	b	SE	*p* Value	b	SE	*p* Value	b	SE	*p* Value
Treatment goal self-efficacy^a^	1.15	1.02	0.260	2.03	0.81	0.013	4.97	1.94	0.011	4.89	2.31	0.037	12.23	3.19	<0.001
Treatment management self-efficacy^a^	0.67	0.93	0.486	2.73	0.74	<0.001	4.89	1.81	0.007	4.57	2.15	0.035	8.58	3.02	0.005
Treatment satisfaction^a^	0.14	0.05	0.005	0.10	0.04	0.023	0.18	0.10	0.076	0.50	0.11	0.001	−0.22	0.17	0.185

^a^Individually adjusted by sex, age, educational level, job, dialysis method, dialysis duration, cause of ESRD, dialysis adequacy, serum albumin level, existence of acute myocardial infarction, ischemic heart disease, stroke, hypertension, diabetes, and number of co-disease

## Discussion

In this study, we measured the HRQOL of dialysis patients, including both HD and PD. In comparison between PD and HD patients, we could find significant difference only in symptom/problem list (i.e., HD patients reported lower QOL than PD patients). There have been several studies using KDQOL-36 for evaluating the HRQOL of patients with chronic kidney disease [25–27], but few studies were conducted in dialysis patients [28]. Compared to QOL results from other studies [25, 28], ours were very similar in PCS, MCS, and Effect of disease. However, some differences were observed in the other domains: We report lower scores in symptom/problem list and higher scores in burden of disease. Restricting to the HD patients, our results were also very similar with the results of the Dialysis Outcomes and Practice Patterns Study [27], which was a prospective, observational, multinational study for HD patients, using KDQOL-SF™.

In our study, dialysis patients showed higher scores in MCS than in PCS, and this has been also reported in several previous studies [25–30]. In other words, despite the worsening of the physical health status, the mental health of dialysis patients is relatively preserved. This was previously explained by dynamic adaptation of patients’ expectations to their chronic illness [11, 30].

We also evaluated whether the self-efficacy and treatment satisfaction, respectively, could impact on the HRQOL of dialysis patients. In this study, after adjusting for the confounders’ effect, self-efficacy on both treatment goal and treatment management showed significant positive association with HRQOL in almost all domains, except PCS. Oh-Park et al. [22] reported positive association between self-efficacy and HRQOL in MCS. Therefore, it remains to be seen whether self-efficacy may influence HRQOL in the generic and disease-specific domains.

Finally, treatment satisfaction was positively associated with HRQOL in PCS, MCS, and effects of disease. These associations are supported by Callahan’s study [23], reporting that patients’ participation in care planning could lead to increased treatment satisfaction and increased HRQOL scores. This implies that high treatment satisfaction can improve patients’ compliance and control disease more effectively, consequently leading to better HRQOL and decrease mortality rate.

Our study has several limitations. First, our participants were volunteer outpatients recruited in two university hospitals; therefore, they had some difference with the general dialysis patients especially in their socio-economic characters and disease severity, and these differences might modified their perception of QOL. Therefore, our results must be applied to general dialysis patients with caution. Second, the instruments, the Korean version of KDQOL-36, self-efficacy and treatment satisfaction questionnaires that we used have not been validated yet.

In spite of those limitations, to the best of our knowledge, this is the first study to use KDQOL-36 for evaluating HRQOL of dialysis patients and to investigate the impact of self-efficacy and treatment satisfaction on HRQOL in Korea.

This study has main findings. First, regular and systematic monitoring for dialysis patients’ HRQOL must be considered as an effective tool of quality control in dialysis care in Korea. Secondly, self-efficacy and treatment satisfaction independently influenced HRQOL in almost all domains. Therefore, interventions to increase self-efficacy and treatment satisfaction should be considered in caring for patients with ESRD.

## References

[1] Hornbrook MC, Goodman MJ (1996). Chronic disease, functional health status, and demographics: A multi-dimensional approach to risk adjustment. Health Services Research.

[2] Konstam V, Salem D, Pouleur H, Kostis J, Gorkin L, Shumaker S (1996). Baseline quality of life as a predictor of mortality and hospitalization in 5,025 patients with congestive heart failure. SOLVD investigations. Studies of left ventricular dysfunction investigators. The American Journal of Cardiology.

[3] DeOreo PB (1997). Hemodialysis patient-assessed functional health status predicts continued survival, hospitalization, and dialysis-attendance compliance. American Journal of Kidney Disease.

[4] Valderrabano F, Jofre R, Lopez-Gomez JM (2001). Quality of life in end-stage renal disease patients. American Journal of Kidney Disease.

[5] Kalantar-Zadeh K, Kopple JD, Block G, Humphreys MH (2001). Association among SF36 quality of life measures and nutrition, hospitalization, and mortality in hemodialysis. Journal of American Society of Nephrology.

[6] Lopes AA, Bragg J, Young E, Goodkin D, Mapes D, Combe C (2002). Depression as a predictor of mortality and hospitalization among hemodialysis patients in the United States and Europe. Kidney International.

[7] Mapes DL, Lopes AA, Satayathum S, McCullough KP, Goodkin DA, Locatelli F (2003). Health-related quality of life as a predictor of mortality and hospitalization: The dialysis outcomes and practice patterns study (DOPPS). Kidney International.

[8] Knight EL, Ofsthun N, Teng M, Lazarus JM, Curhan GC (2003). The association between mental health, physical function, and hemodialysis mortality. Kidney International.

[9] Lopes AA, Bragg-Gresham JL, Goodkin DA, Fukuhara S, Mapes DL, Young EW (2007). Factors associated with health-related quality of life among hemodialysis patients in the DOPPS. Quality of Life Research.

[10] Spiegel BM, Melmed G, Robbins S, Esrailian E (2008). Biomarkers and health-related quality of life in end-stage renal disease: A systematic review. Clinical Journal of American Society of Nephrology.

[11] Singer MA, Hopman WM, MacKenzie TA (1999). Physical functioning and mental health in patients with chronic medical conditions. Quality of Life Research.

[12] Blake C, Codd MB, Cassidy A, O’Meara YM (2000). Physical function, employment and quality of life in end-stage renal disease. Journal of Nephrology.

[13] Chan R, Brooks R, Steel Z, Heung T, Erlich J, Chow J (2011). The psychosocial correlates of quality of life in the dialysis population: A systematic review and meta-regression analysis. Quality of Life Research.

[14] Zrinyi M, Juhasz M, Balla J, Katona E, Ben T, Kakuk G (2003). Dietary self-efficacy: Determinant of compliance behaviours and biochemical outcomes in haemodialysis patients. Nephrology, Dialysis, Transplantation.

[15] Tsay SL, Hung LO (2004). Empowerment of patients with end-stage renal disease-a randomized controlled trial. International Journal of Nursing Studies.

[16] Barendse SM, Speight J, Bradley C (2005). The renal treatment satisfaction questionnaire (RTSQ): A measure of satisfaction with treatment for chronic kidney failure. American Journal of Kidney Disease.

[17] Bandura, A. (1997). Self-efficacy: The exercise of control (pp. 259–313). New York: W.H. Freeman.

[18] Anderson RM, Funnell MM, Fitzgerald JT, Marrero DG (2000). The diabetes empowerment scale: A measure of psychosocial self-efficacy. Diabetes Care.

[19] Kanazawa Y, Nakao T, Ohya Y, Shimomitsu T (2006). Association of socio-psychological factors with the effects of low protein diet for the prevention of the progression of chronic renal failure. Internal Medicine.

[20] Parsons TL, Toffelmire EB, King-VanVlack CE (2004). The effect of an exercise program during hemodialysis on dialysis efficacy, blood pressure and quality of life in end-stage renal disease (ESRD) patients. Clinical Nephrology.

[21] Painter P, Carlson L, Carey S, Paul SM, Myll J (2000). Physical functioning and health-related quality-of-life changes with exercise training in hemodialysis patients. American Journal of Kidney Disease.

[22] Oh-Park M, Fast A, Gopal S, Lynn R, Frei G, Drenth R (2002). Exercise for the dialyzed: Aerobic and strength training during hemodialysis. American Journal of Physical Medicine and Rehabilitation.

[23] Callahan MB (2001). Using quality of life measurement to enhance interdisciplinary collaboration. Advance in Renal Replacement Therapy.

[24] Yildirim A (2006). The importance of patient satisfaction and health-related quality of life after renal transplantation. Transplantation Proceedings.

[25] Zuniga SMC, Dapueto PJ, Muller OH, Kirsten LL, Alid AR, Ortiz ML (2009). Health related quality of life among patients on chronic hemodialysis. Revista Medica de Chile.

[26] Gorodetskaya I, Zenios S, McCulloch CE, Bostrom A, Hsu CY, Bindman AB (2005). Health-related quality of life and estimates of utility in chronic kidney disease. Kidney International.

[27] Fukuhara, S., Lopes, A. A., Bragg-Gresham, J. L., Kurokawa, K., Mapes D. L., Akizawa, T., et al. (2003). Health-related quality of life among dialysis patients on three continents: The dialysis outcomes and practice patterns study. Health-related quality of life among dialysis patients on three continents: The dialysis outcomes and practice patterns study. *Kidney International Enational, 64*(5), 1903–1910. 10.1046/j.1523-1755.2003.00289.x 14531826

[28] Seica A, Segall L, Verzan C, Văduva N, Madincea M, Rusoiu S (2009). Factors affecting the quality of life of hemodialysis patients from Romania: A multicentric study. Nephrology, Dialysis, Transplantation.

[29] Dolan P (1996). Modeling valuations for health states: The effect of duration. Health Policy.

[30] Parsons TL, Toffelmire EB, King-VanVlack CE (2006). Exercise training during hemodialysis improves dialysis efficacy and physical performance. Archives of Physical Medicine and Rehabilitation.

